# Separation and Purification of Sulforaphane from Broccoli by Solid Phase Extraction

**DOI:** 10.3390/ijms12031854

**Published:** 2011-03-10

**Authors:** Dandan Han, Kyung Ho Row

**Affiliations:** Department of Chemical Engineering, Inha University, 253 Yonghyun-Dong, Nam-Ku, Incheon 402-751, Korea; E-Mail: hdd_216@hotmail.com

**Keywords:** broccoli, sulforaphane, solid-phase extraction

## Abstract

A simple solid-phase extraction (SPE) method for the determination of sulforaphane in broccoli has been developed. The optimal conditions were found to be use of a silica SPE cartridge, and ethyl acetate and dichloromethane as washing and eluting solvents, respectively, which could eliminate interferences originating from the broccoli matrix. The extracts were sufficiently clean to be directly injected into high-performance liquid chromatography (HPLC) for further chromatographic analysis. Good linearity was obtained from 0.05 to 200 μg/mL (r = 0.998) for sulforaphane with the relative standard deviations less than 3.6%. The mean recoveries of sulforaphane from broccoli were more than 90.8% and the detection limit (S/N = 3:1) was 0.02 μg/mL. The SPE method provides a higher yield of sulforaphane from crude extracts compared to conventional liquid-liquid extraction.

## 1. Introduction

**Figure 1.**
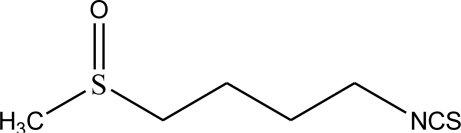
The molecular structure of sulforaphane in broccoli.

Broccoli is a famous vegetable around the world, belonging to the cruciferous family, which is rich in antioxidants such as vitamin C, quercetin and kaempferol [1,2]. Medicinal studies have shown that increasing consumption of broccoli can lower the risk of breast [3], skin [4] and prostate [5] cancers. Recent research showed that glucoraphanin (4-methylsulfinybutyl glucosinolate) is another important constituent in broccoli and it can produces sulforaphane when hydrolyzed by myrosinase. Sulforaphane (Figure 1) has attracted researchers’ attention as a promising cancer chemopreventive agent [6]. In many studies, sulforaphane can reduce the incidence of a number of forms of tumor [7–10]. It is important to develop a rapid and simple method for determination and separation of sulforaphane from broccoli. Several analytical methods such as high-performance liquid chromatography (HPLC) [11,12], GC/mass spectrometry [13] and evaporative light-scattering [14] have been used to determine sulforaphane in broccoli. Meanwhile, preparative HPLC [15] and high-speed countercurrent chromatography [16] have been used to purify sulforaphane. However, the enzymatic hydrolysis of glucoraphanin generates a variety of compounds (glucose, sulfate, isothiocyanates, thiocyanates, nitriles) which interfere with the separation and determination of sulforaphane. Therefore, it is necessary to establish a simple and convenient method for the selective extraction and separation of sulforaphane from broccoli.

In this study, the interference of different hydrolysates was removed by choosing the optimum pH and three different kinds of solid-phase extraction cartridges were used to purify sulforaphane.

## 2. Results and Discussion

### 2.1. Optimization of Chromatographic Conditions

Selection of optimal HPLC conditions is important for the determination and separation of suforaphane. In this study, different wavelengths (205 nm, 235 nm and 254 nm) were investigated, and the results showed sulforaphane had largest absorbance under 205 nm. Therefore, 205 nm was chosen for further analyses.

**Figure 2.**
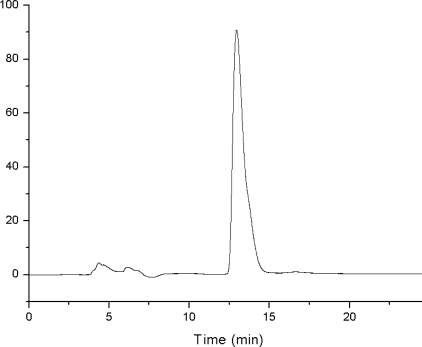
Chromatogram of standard sulforaphane. (Mobile phase: acetonitrile/water (20/80, v/v), injection volume: 10 μL, detection wavelength: 205 nm).

Different kinds of mobile phases such as methanol, acetonitrile, and different concentrations of methanol/H_2_O and acetonitrile/H_2_O were investigated. Under 205 nm, methanol has strong background absorbance, which is difficult to balance the C_18_ column. The 20% acetonitrile/H_2_O (v/v) was proved to provide the best separation, because there is no interference of other impurities and the retention time of sulforaphane is short. The chromatogram of sulforaphane is shown in Figure 2.

### 2.2. Optimization of the Hydrolyzation from Glucoraphanin to Sulforaphane

To optimize the hydrolysis of glucoraphanin to sulforaphane in fresh broccoli, preliminary trials were conducted with different pHs of acidic water (3, 4, 5 and 6), and hydrolysis times (2, 4 and 6 h). The incubation temperature was constant at 35 °C. Table 1 shows that the resulting sulforaphane amount was highest with pH 3.0 and hydrolysis time 4 h.

### 2.3. Optimization of SPE Conditions

#### 2.3.1. Choice of Different SPE Cartridges

In this study, three kinds of SPE cartridges (C_18_, amino and silica) were used, and the elution amount with different solvents are shown in Table 2. These data show that the silica cartridge is better than the other two cartridges for extracting sulforaphane. This may be attributed to the weak polarity of sulforaphane, which be easily selectively absorbed by a weak polarity column.

#### 2.3.2. Influence of the Washing Solvent and Elution Solvent

In the SPE, selection of an appropriate washing solvent and elution solvent is the first factor that should be considered because it has a direct effect on desorption efficiency. Different washing and eluting steps were investigated to optimize the process of selective extraction. It is important to apply a washing step immediately after loading the extract from plants on the sorbent, as it can reduce most of the interference during the separation of the analyte. Initially, different washing solvents with different polarity (water, acetonitrile, dichloromethane and ethyl acetate) were investigated. Table 2 shows that sulforaphane was not washed out by ethyl acetate, while some unnecessary compounds were. Therefore, ethyl acetate was selected as a suitable washing solvent. Moreover, sulforaphane could be washed out simultaneously with a large amount of unnecessary compounds when water, acetonitrile, and dichloromethane were used, where the largest relative amount of sulforaphane was washed out by dichloromethane. Therefore, dichloromethane was used as the elution solvent in subsequent steps.

#### 2.3.3. Influence of Elution Solvent Volume

The volume of elution solvent is another factor that should be considered. A series of experiments were designed and this factor was investigated by changing the volume of the elution solvent from 2 to 6 mL. As shown in Table 3, it can be observed that the extraction amount of sulforaphane increased with increasing volumes of dichloromethane from 2 and 6 mL. When the volume of dichloromethane exceeded 4 mL, the amount of sulforaphane remained almost constant. Therefore, in subsequent experiments, 4 mL of dichloromethane was selected.

#### 2.3.4. Validation of the Proposed Method

A series of standard solutions containing sulforaphane at six concentrations were obtained by mixing the appropriate amount of stock solution (1 mg/mL). Each concentration was analyzed in triplicate. The results are listed in Table 4. The detection limit (S/N = 3:1) was 0.02 μg/mL for sulforaphane, while for other methods it was usually higher than 1 μg/mL [17]. The results showed good precision with a relative standard deviation (RSD) for sulforaphane of 3.54%.

**Figure 3.**
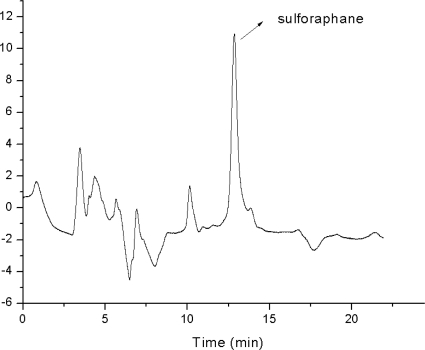
Chromatogram of broccoli extracted by silica-SPE (Injection volume: 10 μL).

The developed SPE-HPLC method was applied successfully to the analysis of sulforaphane from broccoli under the optimum conditions. Figure 3 shows the chromatograms of broccoli when extraction was performed by silica cartidge. Table 5 shows the contents of sulforaphane and recoveries in broccoli.

## 3. Experimental Section

### 3.1. Reagents and Materials

Sulforaphane was obtained from Sigma-Aldrich (USA), and used without further purification. Acetonitrile, methanol, ethyl acetate and dichloromethane were obtained from Duksan Pure Chemical Co., LTD (Ansan, Korea). All the other reagents used in the experiment were HPLC or analytical grade. Double distilled water was filtered with a vacuum pump (Division of Millipore, Waters, USA) and filter (HA-0.45, Division of Millipore, Waters, USA) before use. All the samples were filtered by using a filter (MFS-25, 0.2 μm TF, WHATMAN, USA) before injection into the HPLC system. Stock standard solutions of sulforaphane were prepared by dissolving 10 mg of standards in 10 mL of acetonitrile. Commercial C_18_, amino and silica cartridge (200 mg/3 mL) were purchased from Alltech (USA).

### 3.2. Chromatographic Conditions

Chromatography was performed with a Waters 600 s multisolvent delivery system, a Waters 616 liquid chromatography, and a Waters 2487 variable wavelength, dual-channel, UV detector (Waters Associates, Milford, MA, USA). A six-port Rheodyne injector (20 μL sample loop) was also used. Data processing was performed with Millennium 3.2 software resident in an HP Vectra 500PC. Compounds were separated on a 250 mm × 4.6 mm, 5-μm particle, OptimaPak C_18_ column (RS Tech, Daejeon, Korea). HPLC separation of sulforaphane was conducted by using acetonitrile/H_2_O (20/80, v/v) as mobile phase at a flow rate of 0.5 mL/min and the detection was carried out at a wavelength of 205 nm. Distilled water was filtered with a vacuum pump and filter (HA-0.45 μm; Millipore, Waters, USA) before use.

### 3.3. Sample Clean-up and Preparation by SPE

Fresh broccoli was pulverized and 5 g of the resultant powder was weighed and extracted with 20 mL of different pH of hydrochloric acid (HCl) for 2 h. The resulting mixture was extracted 3 times with 20 mL of dichloromethane, which was combined and salted with anhydrous sodium sulfate. The dichloromethane fraction was dried at 30 °C under vacuum on a rotary evaporator. The residue was dissolved in acetonitrile and was then filtered through a 0.22 mm membrane filter for the following study. The sulforaphane was purified with different SPE cartridge. Prior to use, the silica cartridge was conditioned with 4 mL of dichoromethane, the C_18_ cartridge was conditioned with 4 mL methanol and amino cartridge was conditioned with 4 mL ethyl acetate. First, 200 μL of organic extract was loaded through the three different kinds cartridges, then the silica cartridge was washed with 4.0 mL of ethyl acetate (which was then discarded) and the sulforaphane eluted with 4 mL of dichloromethane; the C_18_ cartridge was washed with 4 mL of water (which was then discarded) and the sulforaphane eluted with 4 mL of 0.1 mol/L of acetic acid; and the amino cartridge was washed with 4 mL of ethyl acetate (which was then discarded) and the sulforaphane eluted with 4 mL dichloromethane. The obtained extractants were evaporated to dryness in a vacuum oven at 45 °C for 2 h, and redissolved with 2 mL of acetonitrile. The resulting solutions were vortexed for 30 s and filtered with a membrane of 0.45 μm. 10 μL samples of these solutions were then injected into the column of the HPLC system. All samples were analyzed in duplicate.

## 4. Conclusions

A simple and sensitive SPE-HPLC assay procedure was developed for the extraction and determination of sulforaphane from broccoli. The absorption wavelength was determined at 205 nm. The elution mobile phase was ACN/H_2_O (20:80, v/v). Compared with C_18_ and amino cartridges, the silica cartridge displayed better selectivity to sulforaphane. Ethyl acetate was used as a washing solvent, and 4 mL of dichloromethane as an elution solvent. The extracted amount of sulforaphane from broccoli was 0.513 mg/g.

## Figures and Tables

**Table 1.** The amount of extracted sulforaphane under different pH conditions.

**pH**	**Amount of sulforaphane (mg/g)**
0	0.024
2.0	0.057
3.0	0.148
4.0	0.132
6.0	0.118

**Table 2.** Extracted amounts of sulforaphane when using different solvents in the washing step and with different cartridges.

**Washing solvent**	**Amount of sulforaphane (mg/g)**		
	**Silica cartridge**	**C_18_ cartridge**	**Amino cartridge**
Water	0.016	0.003	0.002
Acetonitrile	0.069	0.032	0.027
Dichloromethane	0.357	-	0.057
0.1M Acetic acid	-	0.062	-
Hexane	-	-	0.006
Ethyl acetate	0	0	-

“-”: not used in the washing step.

**Table 3.** Extracted amounts of sulforaphane by different volumes of dichloromethane in the elution step (Silica cartridge).

**Volume of dichloromethane (mL)**	**Amount of sulforaphane (mg/g)**
2	0.120
3	0.214
4	0.324
5	0.326
6	0.327

**Table 4.** Calibration range (n = 5) and LOQ for the quantification of sulforaphane.

**Target compound**	**Linear range (μg/mL)**	***r*^2^**	**LOD (μg/mL)**
Sulforaphane	0.05–200	0.998	0.002

**Table 5.** Recovery of sulforaphane in three concentration levels.

	**Original (μg/mg)**	**Add amount (μg/mg)**	**Found amount (μg/mg)**	**Recovery (%)**	**RSD**
Sulforaphane	0.513	10.0	9.59	90.8	3.54
	0.513	50.0	47.10	93.2	3.17
	0.513	100.0	96.91	96.4	2.36
